# Mixed‐Polymer Hole Transport Layers Reinforce Au‐Anode Top Emitting Perovskite Light Emitting Diodes

**DOI:** 10.1002/advs.76051

**Published:** 2026-06-11

**Authors:** James C. Loy, Jisu Hong, Tuo Hu, Xu He, Antoine Kahn, Barry P. Rand

**Affiliations:** ^1^ Department of Physics Princeton University Princeton New Jersey USA; ^2^ Department of Electrical and Computer Engineering Princeton University Princeton New Jersey USA; ^3^ Andlinger Center for Energy and the Environment Princeton University Princeton New Jersey USA

**Keywords:** anode oxidation, electrochemistry, halide, iodide, materials science, metal halides, methylammonium lead halide, optoelectronics, perovskite

## Abstract

As metal halide perovskite light‐emitting diodes (PeLEDs) rapidly improve in performance, identifying and resolving factors that limit operational stability is paramount. Compounds released by the perovskite as byproducts of interfacial electrochemistry can react with and compromise various LED layers and diminish performance, particularly corroding and degrading metal anodes. In this study, we use a mixed‐polymer hole transport layer (a mixture of poly(N,N'‐bis‐4‐butylphenyl‐N,N'‐bisphenyl)benzidine (polyTPD) and poly(9‐vinylcarbazole) (PVK)) to stabilize methylammonium lead iodide‐based PeLEDs with Au metal anodes against increasing leakage current under repeated operation and decreased emission intensity under constant driving. We study this layer using a newly developed Au‐corrosion‐based triiodide detection experiment to determine that our mixed hole transport layer interferes with and inhibits triiodide transport, leading to more stable operation.

## Graphical abstract

Au‐anode corrosion is shown to occur rapidly under voltage bias in MAPbI_3_ perovskite LEDs. Resulting Au^+^ migration quickly leads to device shorting and unstable emission during constant current driving. We demonstrate that using a simple mixed‐polymer hole transport layer containing polyTPD and PVK can reduce Au‐corrosion and stabilize LED operation without reducing hole‐injection efficiency.

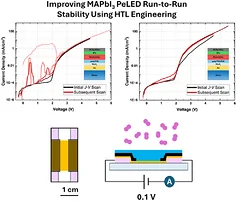

## 1 Introduction

Metal halide perovskites have been heavily studied over the last decade as next‐generation light‐emitting diodes (LEDs) for their high color purity and tunability as well as their potential for low cost of manufacture [1]. Their performance has rapidly advanced during this time, reaching external quantum efficiencies (EQEs) of over 20% across the visible and near‐infrared (NIR) spectrum [2, 3, 4, 5, 6]. Despite these gains, commercial adoption of perovskites must overcome issues of stability that vary with different perovskite formulations and device structures.

Perovskite LED (PeLED) stability encompasses issues ranging from halide phase separation to EQE roll‐off at high current densities to device degradation under intense or persistent electrical driving. The latter is due to physical damage to the LED structure and can be made apparent via run‐to‐run variation in performance, such as increased leakage current and changes in electroluminescence (EL) intensity at a given voltage. Metal halide perovskites consisting of potentially redox‐active compounds have been shown to leach reactive species into LED support layers, particularly the surrounding charge transport layers and metal electrodes. The most salient example involves halide species, which oxidize a metal electrode in the LED stack, requiring careful separation between the perovskite layer and the (typically Al or Ag) contact electrodes [7]. Replacing the electrodes with a more chemically inert metal, such as Au, can improve the repeatability of PeLEDs, but iodine‐based degradation of the Au electrode [8] can still lead to run‐to‐run instabilities that make the PeLED impossible to characterize. This is particularly a problem for p‐i‐n top emitting PeLEDs where a reflective metallic anode is useful to inhibit substrate losses.

Top emitting PeLEDs reverse the standard emission direction to avoid transmitting through the substrate. Instead, emission occurs through a semi‐transparent top‐electrode, removing the substrate from the optical path entirely [9, 10]. This has many potential benefits from preventing optical losses in thick substrates, maintaining coherent emission, and utilizing a microcavity to promote radiative decay in the perovskite layer via the Purcell effect [11]. Perhaps one of the biggest benefits is the ability to utilize opaque substrates that can offer higher thermal conductivities. As perovskites are very sensitive to thermal damage from Joule heating, replacing traditional transparent glass and sapphire with more effective heat‐sink materials like Si can improve long‐term operation of LEDs, and may ultimately be the only way to thermally manage future electrically pumped perovskite lasers [12, 13]. Despite these benefits, top emitting PeLEDs are relatively rarely reported in literature, due to the simplicity offered by using a commercially‐prepared and relatively robust transparent conducting oxide on a transparent substrate to guarantee an efficient emission pathway. Additionally, top emitting PeLEDs require a reflective electrode on the substrate to prevent significant losses. This is easiest achieved using a thick reflective metal electrode, but if a p‐i‐n architecture is used, the reflective metal anode will be subject to halide corrosion. If an n‐i‐p architecture is used instead, the thin semi‐transparent anode remains susceptible to corrosion. Achieving a stable top‐emitting PeLED therefore requires the anode to be protected by the supporting layers in the device. If the charge transport layers (CTLs) prevent (or limit) halide transport through the LED, degradation can be avoided [8]. Deep HOMO materials are known, for instance, to block halide migration by being energetically unfavorable to oxidize [14]. However, for an efficient PeLED, the need for proper band alignments and conductive interfaces limits which CTL materials can be used.

In this work, we observe degradation‐based instability in top emitting PeLEDs that rely on Au‐anodes with polyTPD hole transport layers (HTLs). We identify halide‐based corrosion of the metal anode as the source of the issue. We propose a simple‐to‐fabricate mixed‐polymer HTL that interferes with this degradation mechanism and demonstrate the stabilizing effect our mixed‐polymer HTL has on PeLED performance. We then characterize the layer and find direct evidence that our mixed‐polymer film interferes with a triiodide‐iodide lixiviant reaction at the anode surface as the mechanism for the improved performance.

## 2 Results and Discussion

**FIGURE 1:**
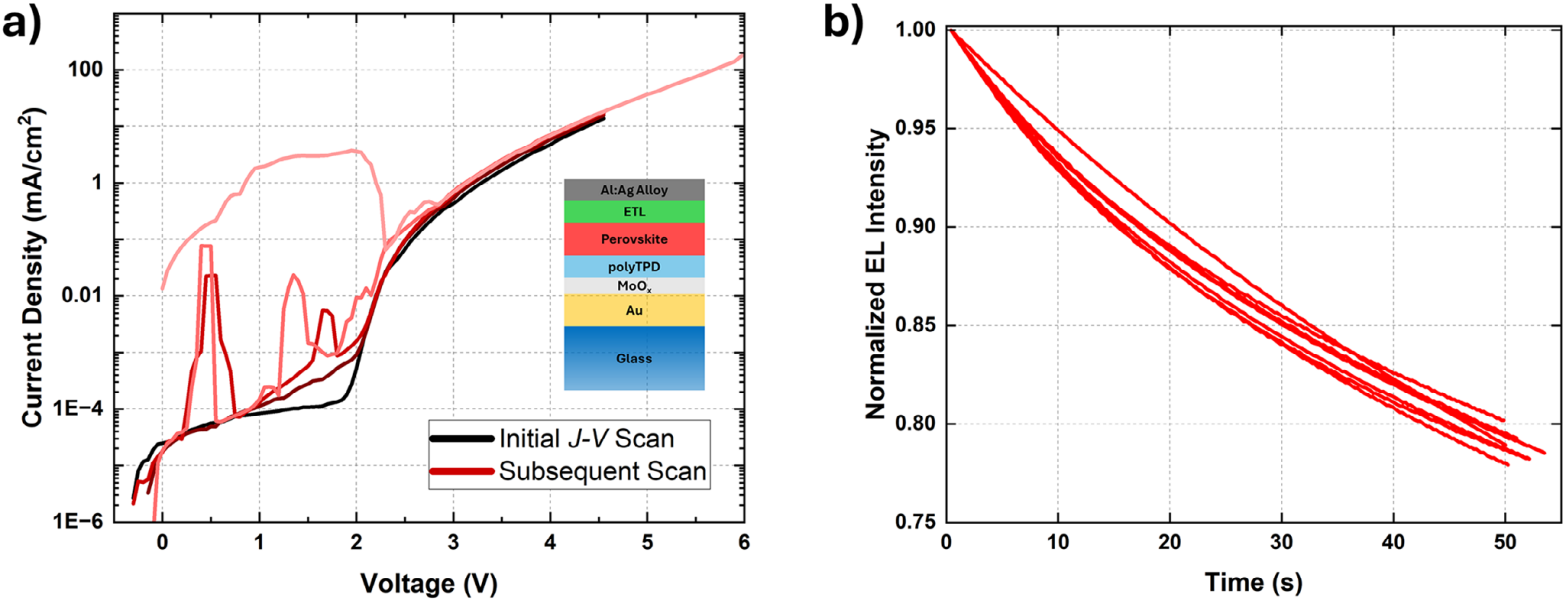
(a) Top emitting perovskite LED *J–V* characteristics under repeated voltage sweep. Pixels rapidly increase in leakage current under repeated operation. Consecutive LED operation shown by increasingly lighter red scans (5 total sweeps are displayed from a single representative pixel). (b) Under constant operation at a fixed current density of 5 mA cm^−2^, emission intensity drops significantly within 50 s. Each trend represents a distinct but nominally identical pixel.

For perovskite LEDs that rely on metallic anodes, halide‐induced corrosion is a significant barrier to their reproducibility and operational stability. Figure 1a shows current density vs. voltage (*J–V*) characteristics from a top emitting PeLED upon repeated operation over a range of driving voltages. Each dataset scan is a voltage sweep from −0.5 V to a (variable) maximum voltage in steps of 0.05 V with roughly 0.1 s wait time at each step. The initial sweep shows very low leakage current below the turn‐on voltage near 2 V, with a clear diode‐like curve. Subsequent measurements of the same pixel show an increasing leakage current initially appearing as inconsistent spikes in leakage current before evolving into persistent leakage currents with a similar magnitude to the diode's maximum current density. This behavior appears regardless of the maximum voltage of the previous operation, indicating it is not due to perovskite layer degradation caused by excessive driving voltage. In addition to leakage spikes, we observe a coincidental issue in top emitting PeLEDs where sustained current driving results in unstable emission during operation. Using photocurrent as a proxy, we found that top emitting PeLED pixels lose 15%–25% of their initial emission intensity over the course of 50 s (see Figure 1b). Both issues are absent in bottom emitting PeLEDs when the anode consists of indium tin oxide (compare with Figure S1, where leakage current is consistently lower and EL intensity stays within 5% of the initial), suggesting a deleterious reaction unique to metallic anodes.

The devices used in Figure 1 pair a methylammonium lead iodide (MAPbI_3_) perovskite emitting layer with a gold metal anode. Our LEDs reach maximum current densities of roughly 100 mA/cm^2^ and maximum luminance of 1000 cd/m^2^ at 6 V, achieving a peak external quantum efficiency of just over 9.5% at 20 mA/cm^2^, falling only to 9% at 100 mA/cm^2^. Emission is in the NIR region (765 nm peak) and is strongly forward biased due to the microcavity formed by the pair of metal electrodes framing the LED stack. While Au is a popular anode material due to its relative chemical inertness, studies have identified that iodine can lead to Au corrosion when reduced to iodide and triiodide species in aqueous solutions [15]. Kerner et al. were able to show that Au oxidation and liberation occur even in solid state devices when the HTL facilitates iodine reduction and allows transport of reactive species from an iodine‐rich perovskite layer to a nearby Au anode [8]. Further, liberated Au migration throughout the device was shown to occur under a relatively mild voltage bias of < 1.5 V over distances far exceeding our LED's perovskite layer thickness of 30 nm [8, 16]. We therefore propose that top emitting LED device operation at considerably higher voltages induces rapid Au corrosion, leading to the degradation symptoms displayed in Figure 1. Initial LED operation consistently exhibits low initial leakage current, but the applied voltage bias oxidizes and releases iodine‐containing species from the MAPbI_3_ layer, such as I_2_, I^0^, and HI (which can decompose to I_2_) [14]. These products are reduced by our HTL to iodide (I^−^) and triiodide (I_3_^−^) during repeated operation. A lixiviant reaction subsequently allows oxidation of Au in the presence of triiodide and iodide into gold (I) diiodide (2Au + I_3_^−^ + I^−^ → 2[AuI_2_]^−^) [17]. These products can dissociate back into liberated Au^+^ cations, which can diffuse/drift into the rest of the LED, electronically altering the perovskite layer [16, 18], decreasing shunt resistance, and interfering with charge balance. Mobile Au^+^ cations can also be reduced to Au^0^ at the cathode side of the LED, seeding possible shunting locations that can develop during LED driving [16]. We propose two possible mechanisms that generate the run‐to‐run instabilities observed. First, Au deposits may form continuous metal filaments throughout the LED that provide high conductivity, non‐radiative pathways connecting the anode to the electron transport layer and/or cathode directly. These would lead to spontaneously formed high‐current pathways, which, at first, quickly burn out from Joule heating. This would explain the initial leakage current spikes that appear suddenly but disappear quickly as the LED is operated to higher bias voltages (see Figure 1a). Over repeated driving, filaments are reinforced with more liberated Au^+^ until persistent conductive pathways bypass the heterojunction entirely, leading to larger leakage current spikes and, ultimately, to completely shorted devices. Second, Au deposits may build up in the perovskite layer itself with repeated driving, increasing the conductivity of the emission layer and creating more sites for nonradiative recombination. The process appears to occur very quickly, shorting out most diodes within five voltage sweeps. Under constant current driving, a modest current is maintained using a voltage just above turn‐on, during which the Au migration process continues and appears to reduce the effective voltage drop over the perovskite, leading to reduced emission intensity. Alternatively, the increase in nonradiative recombination centers from Au deposits reduces the perovskite's internal quantum efficiency. It is possible other voltage‐based MAPbI_3_ degradation mechanisms may be occurring during operation. However, we expect these mechanisms to be equally present in our bottom emitting control device. As our control does not show either degradation/instability signal seen in top emitting PeLEDs (compare Figure 1 and Figure S1), we reasonably conclude these other mechanisms are likely not at fault. Other iodine‐sensitive metals in our LED stack, specifically Al and Ag in our cathode, are not a source of instability as they are also safely present in the bottom emitting control, and cathodically biased metals are not at risk of oxidation.

**FIGURE 2:**
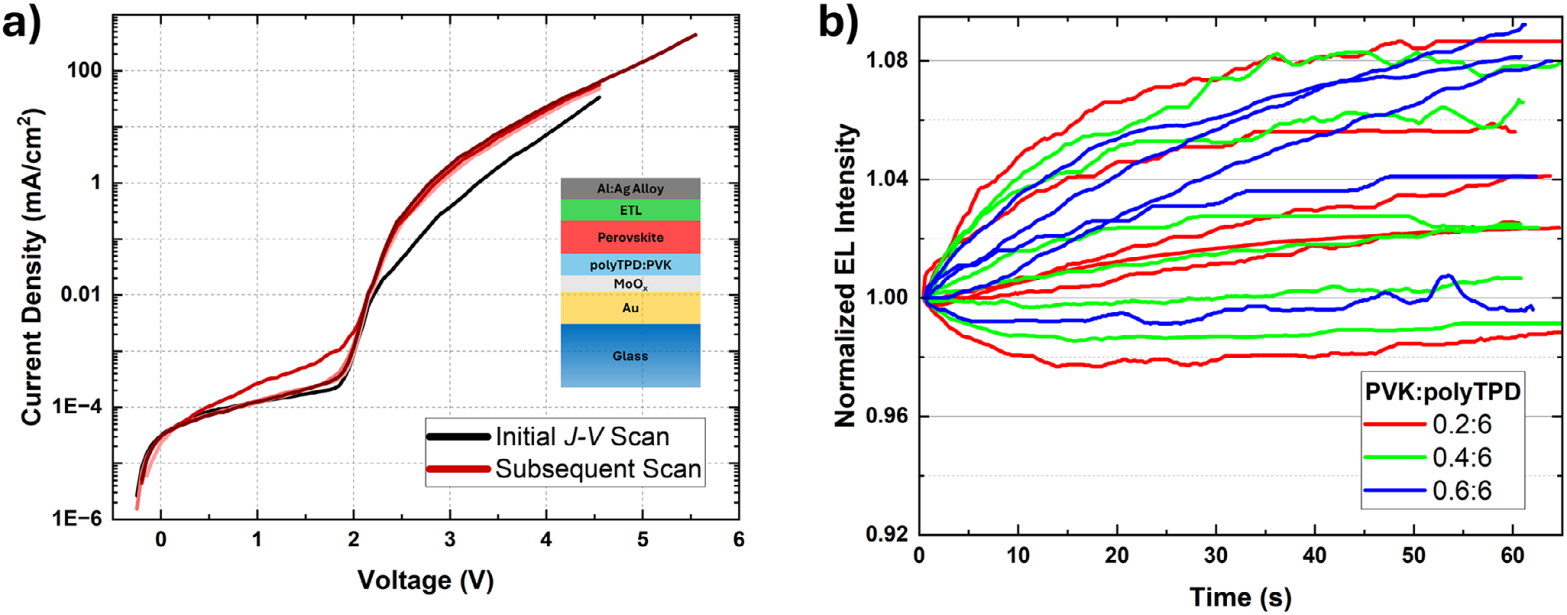
(a) Top emitting perovskite LED *J–V* characteristics under repeated operation with a mixed PVK:polyTPD halide blocking HTL (0.4:6 weight ratio). Consecutive LED operation is shown by increasingly lighter red scans (5 total sweeps are displayed from a single representative pixel). (b) Normalized EL intensity under constant operation at a fixed current density of 20 mA cm^−2^ is maintained above 96% of the initial intensity over the course of 60 s for LEDs with various ratios of polyTPD and PVK. Legend labels specify the weight ratios of the polymers per mL of solvent.

To stabilize the LED against our theorized failure mechanisms, we undertook efforts to reduce triiodide formation in the HTL. Iodine transport across solid state organic HTLs is governed in part by the highest occupied molecular orbital level (HOMO) of the material: deeper HOMO level hole transport materials can suppress iodine uptake and migration [14, 19, 20]. A commonly used polymeric HTL for MAPbI_3_ is poly(N,N'‐bis‐4‐butylphenyl‐N,N'‐bisphenyl)benzidine (polyTPD) owing to its suitable band alignment with the perovskite valence band and its intermediate HOMO level (5.2 eV) [21]. Unfortunately, this material is readily doped by iodine and promotes rapid triiodide formation [14]. Poly(9‐vinylcarbazole) (PVK) is known to interfere with iodine transport in an LED due to its deep HOMO level (5.8 eV) [22], but its inclusion in a MAPbI_3_ LED drastically increases the series resistance of the device as it introduces a barrier for hole injection. Efforts to use a bilayer HTL with polyTPD and PVK are rare due to the strong overlap of their respective solvents, inevitably damaging whichever polymer serves as the underlayer in solution‐based fabrication. Chen et al. reported using heated m‐xylene for PVK as an orthogonal solvent to polyTPD for use in quantum dot LEDs with bilayer HTLs [23], but we are unable to reliably reproduce their bilayer result in our laboratory. Attempts at protecting deposited polyTPD by promoting cross‐linking at the surface via annealing before PVK deposition were similarly unreliable. Instead, we found that forming a co‐solution of the two polymers in the same solvent (chlorobenzene, CB) created a mixed‐polymer HTL capable of maintaining efficient hole injection into our perovskite while interfering with iodine species transport. Figure 2 shows the notable effect small amounts of PVK have on PeLED performance (representative LED characterization data for these stabilized PeLEDs are shown in Figure S2). Leakage current is heavily suppressed for repeated operation, and photocurrent output is maintained above 96% for at least 60 s of operation, which is sufficient time to accurately characterize LED performance. Furthermore, the mixed HTL devices show similar current levels and turn‐on voltage when compared to the neat polyTPD devices, demonstrating efficient hole injection with our mixed HTL.

Our mixed‐polymer co‐solution was found to have a narrow window of solubility due to poor co‐solubility at high concentrations. For concentrations of 6 mg/mL of polyTPD in CB, adding more than 1 mg/mL of PVK led to a viscous gel‐phase that prevented uniform film deposition, but for solutions with less than 1 mg/mL PVK (6 mg/mL polyTPD held constant), the visible viscosity was comparable to that of non‐mixed HTL solutions and led to smooth, uniform films of comparable thickness that promoted high‐quality perovskite growth (see Table 1 for layer thicknesses for mixed HTL films, as well as exact concentrations used due to how mixed polymer film solutions were combined, see Methods section 4.1 for details). We found that a target concentration of 0.4 mg:6 mg:1 mL PVK:polyTPD:CB was ideal for repeatable, stable top emitting perovskite LEDs with Au anodes. Our fabrication method has the benefit of simplicity, avoiding potential underlayer damage that can occur when attempting to make bilayer films; a single spin‐coat deposition and anneal process completes the HTL formation without the need for exotic or heated solvents. Atomic force microscopy (AFM) images of the film surfaces with measured roughness characteristics are shown in Figure S3. All films are remarkably smooth and show no significant pinholes across the films (measured roughness was below 1 nm for all samples). A slight increase in surface roughness is visible for the mixed films, with higher quantities of PVK correlating with increased roughness. This is consistent with the increased viscosity observed for the mixed‐polymer solutions with higher PVK loading.

**TABLE 1:** Translation to exact ratios of material in mixed HTL films and their standalone layer thicknesses as measured by ellipsometry.

HTL Material Label	Target Concentration	Exact Concentration	Layer Thickness
polyTPD	6 mg/mL	6 mg/mL	18 nm
PVK	6 mg/mL	6 mg/mL	24 nm
“0.2 PVK”	0.2:6 mg/mL	0.19:5.81 mg/mL	20 nm
“0.4 PVK”	0.4:6 mg/mL	0.37:5.62 mg/mL	22 nm
“0.6 PVK”	0.6:6 mg/mL	0.55:5.45 mg/mL	24 nm
“0.8 PVK”	0.8:6 mg/mL	0.71:5.29 mg/mL	—
“1.0 PVK”	1.0:6 mg/mL	0.86:5.14 mg/mL	27 nm

**FIGURE 3:**
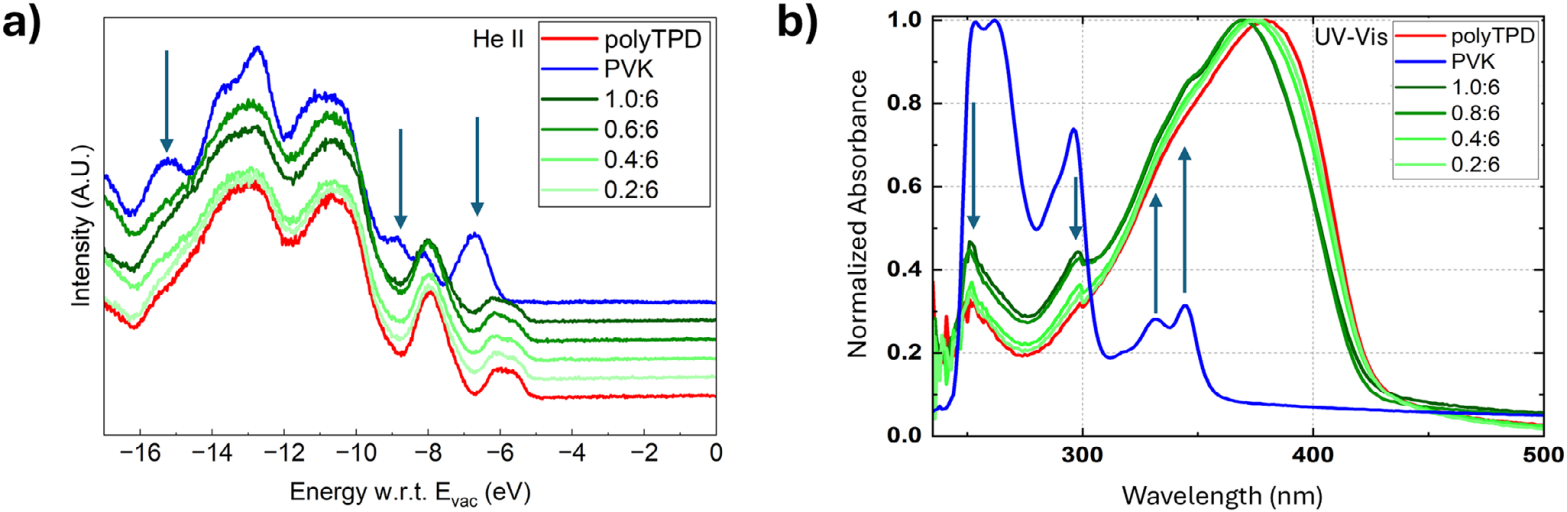
Spectrographic analyses of the polymer HTL films. (a) He II UPS spectra of neat PVK, polyTPD, and mixed‐polymer films. The density of states (DOS) shows unique features for PVK compared to polyTPD (arrows). None of these features appear in any mixed film DOS, suggesting nearly pure polyTPD top surfaces. (b) UV–Vis absorption spectra of the various HTL films. PVK and polyTPD are distinguishable by their absorption spectral peaks. In our mixed films, increasing PVK loads show characteristic PVK peaks atop the predominantly polyTPD spectra (arrows), indicating PVK incorporation within the bulk and/or at the buried interface of the complete films.

To analyze our mixed‐polymer HTL we performed a few characterization measurements. Figure 3 plots spectrographic analyses of the polymer films used in our study. Figure 3a shows the valence states of the HTL measured by ultraviolet photoelectron spectroscopy (UPS) using He II radiation. No features unique to PVK (indicated by arrows) appear in the density of states (DOS) for any mixed film, with peak features appearing unchanged from the polyTPD DOS. Additionally, He I UPS data reveal polyTPD has a distinctly shallower HOMO level than PVK (0.8 and 1.4 eV below Fermi level (E_F_), respectively), and all mixed‐polymer films strongly favor a purely polyTPD makeup in their measured HOMO levels (0.7 eV below E_F_ for all films, see He I data in Figure S4). Further, ionization energies strongly favor the surface being enriched in polyTPD (5.9 eV for PVK, 5.1 eV for polyTPD, 5.1 eV for all mixed films, see Table S1. All preceding UPS energies were measured to ±0.1 eV resolution). Since UPS is most sensitive to the surface of the polymer layer, this suggests that at the perovskite/HTL interface the HOMO level of the mixed film is unchanged with respect to that of polyTPD, and the film is dominantly or completely polyTPD in makeup even for the heaviest PVK inclusion. Contact angle measurements further support the conclusion that the surface makeup of our mixed films is dominated by polyTPD (see Figure S3c). This is consistent with the mixed films’ similar electronic function as effective HTLs in the PeLED. The HTL‐perovskite interface (and therefore hole injection) is virtually unchanged for mixed‐polymer devices compared to the pure polyTPD HTL control, which explains why the *J‐V‐L* performance is not negatively impacted. This also suggests that crystallization of the perovskite at this interface is unaffected by the addition of PVK and therefore not a contributor to the stabilization we observe. This is important as modifying HTLs with ultrathin layers at the perovskite boundary has been shown to influence perovskite wettability and crystallization, leading to operational stability improvements [20].

Figure 3b shows UV–Vis absorbance spectra, which sample the bulk of the polymer layer. Pure polyTPD and PVK films have distinct absorbance spectra in the ultraviolet (UV) to visible range, with polyTPD showing a much wider absorption bandwidth than PVK in the near‐UV range. The presence of PVK is confirmed in our annealed mixed films as shoulder features appear between 250 and 350 nm in the mixed film absorbance spectra corresponding to absorption peaks in the PVK spectrum (see arrows in Figure 3b). These shoulder features are more pronounced for films with higher ratios of PVK, consistent with higher loadings.

**FIGURE 4:**
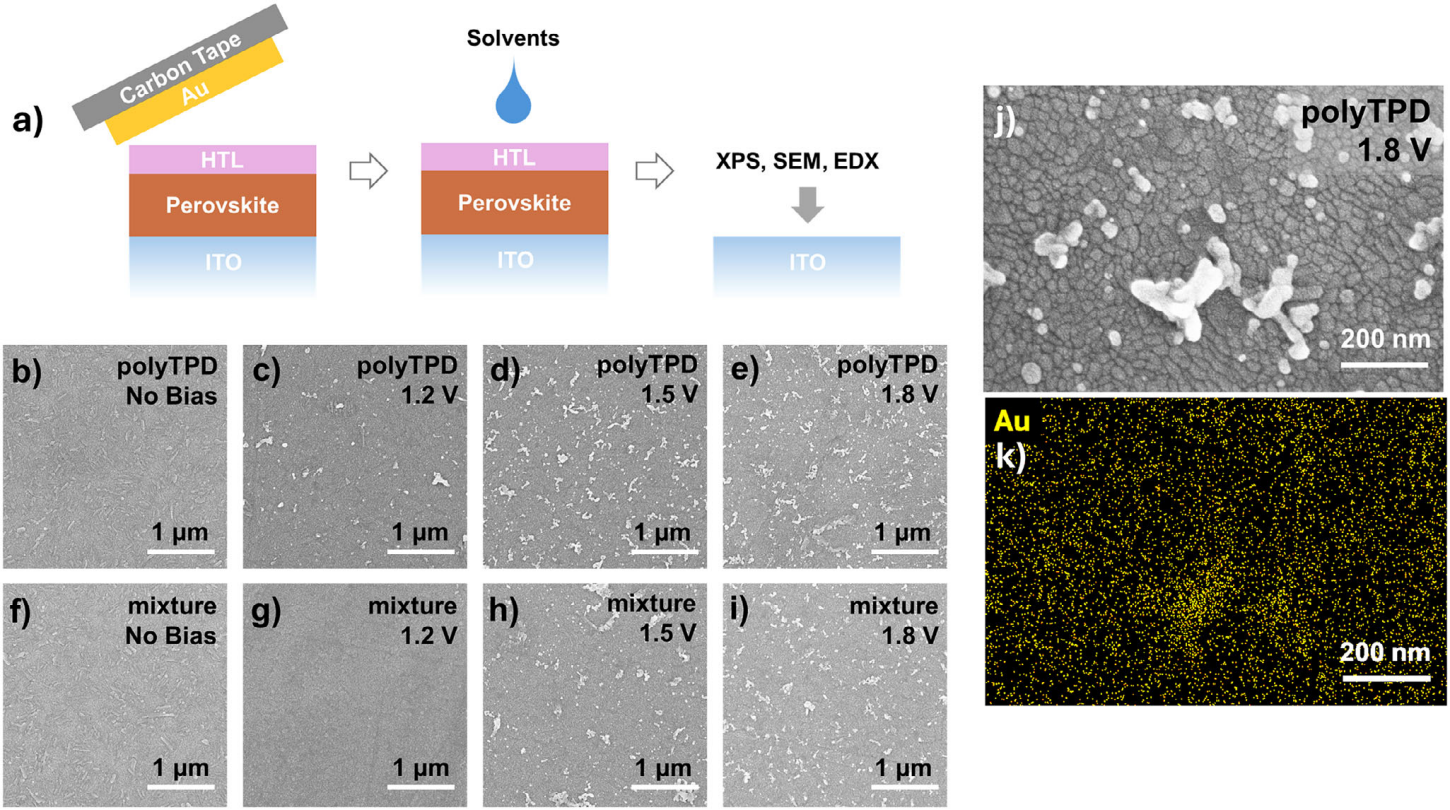
Au‐migration test procedure and results. (a) Schematic of half‐stack LED samples showing Au‐anode removal and exposure of the ITO cathode for SEM, EDX, and XPS (see Figure 5). (b–i) Scanning electron microscopy (SEM) images of the exposed ITO interface from devices with (b–e) polyTPD or (f–i) mixed‐polymer (0.4:6 PVK:polyTPD) HTL biased at the specified voltage for 2 h. (j) Zoom‐in SEM image of the bright islands deposited on the exposed ITO surface, together with (k) the corresponding energy dispersive x‐ray spectroscopy (EDX) mapping for Au (brightness adjusted for clarity).

**FIGURE 5:**
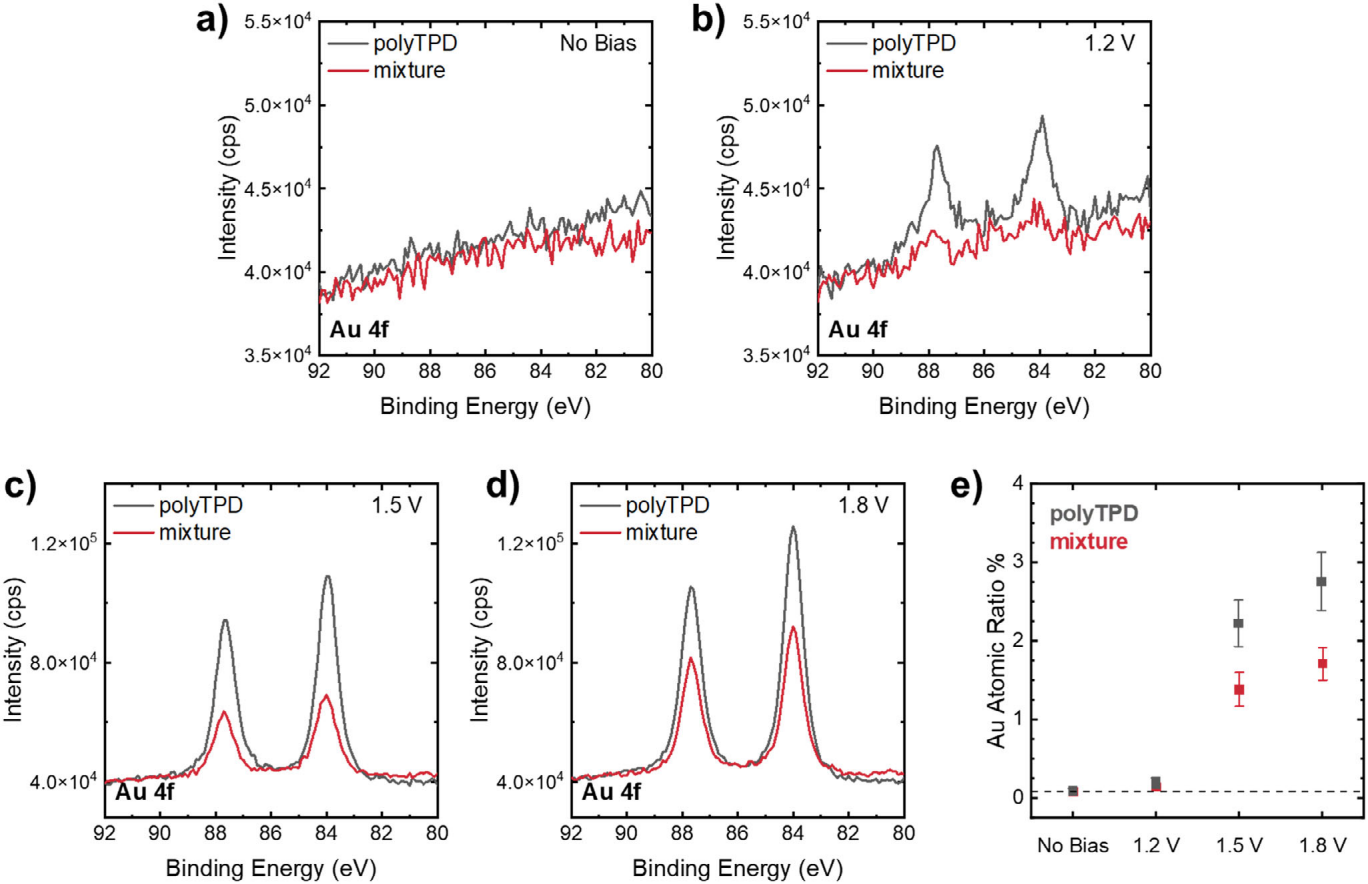
Au 4f XPS spectra of the exposed ITO interface from devices with polyTPD or mixed‐polymer (0.4:6 PVK:polyTPD) HTL biased at (a) 0 V, (b) 1.2 V, (c) 1.5 V, and (d) 1.8 V for 2 h. (e) Au atomic percentages, defined as the ratio of Au atoms to the total number of atoms detected within the XPS measurement volume, as a function of applied voltage. Error bars in (e) reflect the standard deviation of Au atomic percentages measured by XPS.

We then confirmed that Au migrates through our PeLEDs when electrically biased by isolating the hole‐side of our LEDs and utilizing SEM, energy‐dispersive x‐ray spectroscopy (EDX), and x‐ray photoelectron spectroscopy (XPS) measurements. Figure 4a shows our experimental devices and process. Half‐devices with a structure of ITO/MAPbI_3_/HTL/Au were fabricated and biased with various voltage levels for 2 h for pure polyTPD and for the “0.4 PVK” mixed film. Perovskite films were 30 nm thick. Voltage levels were chosen based on previous similar work by Kerner et al. [8]. Au was delaminated using carbon tape, with HTL and perovskite layers removed via solvent treatments. Under zero bias, no aggregation was detected at the cathode layer for either pure polyTPD or the mixed film (Figure 4b,f). For 1.2 V of bias, pure polyTPD showed deposited islands on the ITO cathode while the mixed film was unchanged (Figure 4c,g). At 1.5 V, both films showed scattered island‐like deposits with the mixed‐polymer HTL showing visibly lower deposit density compared to pure polyTPD (Figure 4d,h). This trend continued for 1.8 V bias with higher deposit density for both films (Figure 4e,i). An SEM closeup on one of these island growth sites (Figure 4j) shows they overlap with dense clusters of Au detected in the EDX measurement (Figure 4k). These data serve as direct observation of liberated Au migrating all the way through the perovskite layer and collecting on the cathode layer. The mixed layer HTL clearly interferes with this degradation, raising the onset voltage of the Au accumulation in the cathode and slowing the rate of transport at higher biases. To quantify Au accumulation in the cathode, Figure 5 shows XPS data collected on the ITO cathode surface for each of the samples examined in Figure 4b–i. The detection of Au 4f peaks in the XPS results reflects the visual trend in the SEM images. Under no bias, no Au 4f peaks appear on the ITO electrode, with the signal at baseline noise (Figure 5a). At 1.2 V, a small signal shows Au is starting to accumulate at the cathode for the pure polyTPD HTL, while the mixed film shows essentially baseline noise (Figure 5b). At 1.5 and 1.8 V Au 4f peaks are visible in both samples (Figure 5c,d), but the mixed film's Au signal intensity remains significantly lower compared to the level for neat polyTPD. Au atomic ratio % is calculated in Figure 5e for each applied bias and shows that even for high electrical bias, the mixed film remains around 60% of the polyTPD sample's saturation ratio for the same bias and time. Taken together, these data provide direct evidence for Au‐migration in our PeLEDs, as well as the reduction of accumulation when a mixed‐polymer HTL is used in lieu of pure polyTPD. The findings are completely consistent with our hypothesized degradation mechanism and the improved stability of our top emitting PeLEDs.

**FIGURE 6:**
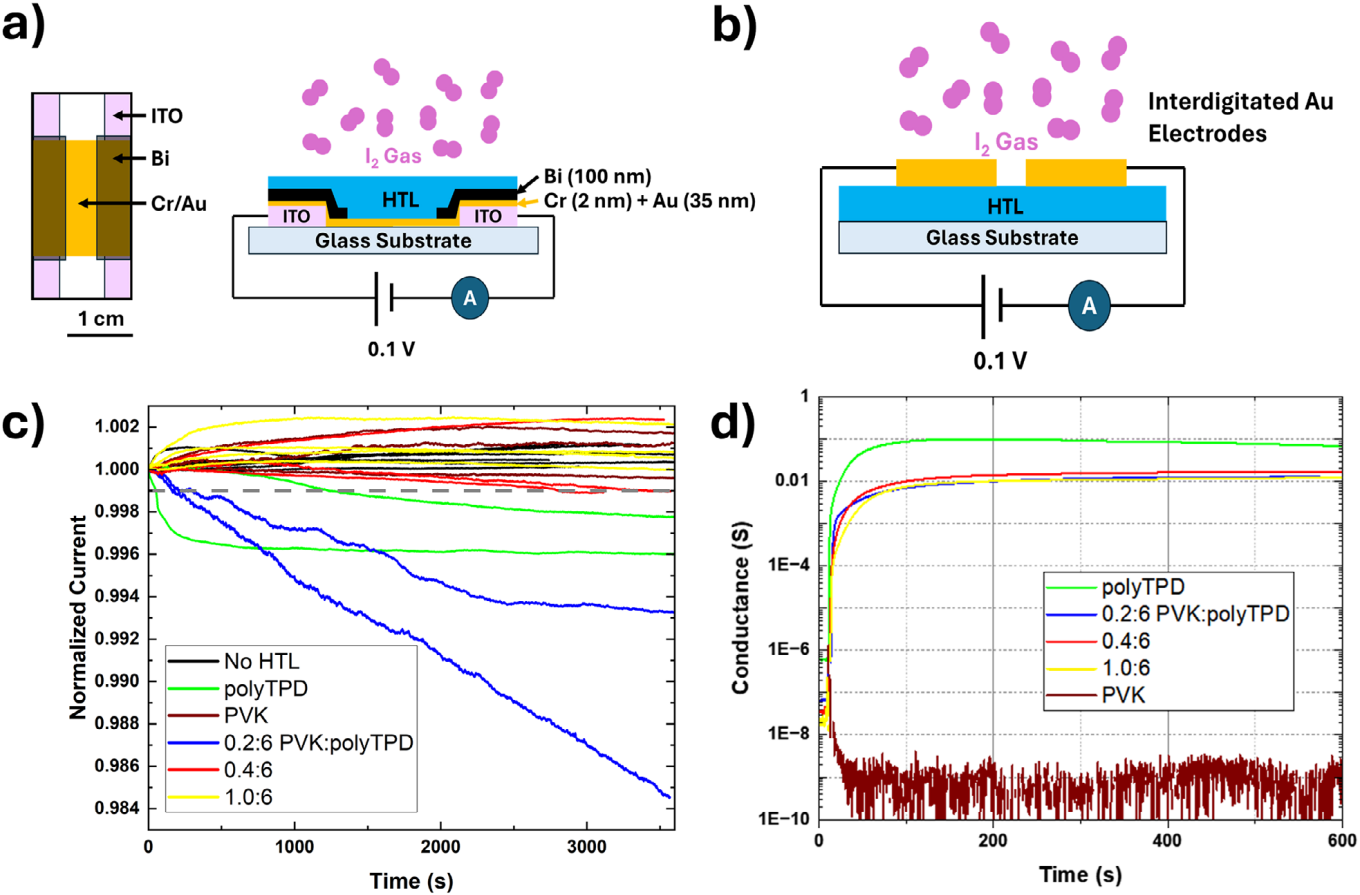
(a) Au‐fuse test sample structure. A thin film of Au is exposed to a saturated I_2_ atmosphere through a uniform window defined by evaporated Bi layers. HTL materials deposited on top can interfere with iodine‐based corrosion at the fuse surface. (b) Conductance test samples. Au electrodes test the conductivity through the HTL as I_2_ gas is allowed to infiltrate the film. Closely spaced (150 µm) and interdigitated electrodes detect increases in film conductivity in response to iodine doping. (c) Normalized current vs. time for various HTL samples in an I_2_ ambient. The gray dashed line at 99.9% initial current level serves as a guide to the eye. (d) Conductivity test for HTL films under I_2_ exposure. Pure PVK shows no I_2_ interaction and thus no change in conductivity. Samples with polyTPD show a significant increase in current, with saturation occurring an order of magnitude below that of pure polyTPD when PVK is present.

We have established that Au migration occurs in our pure polyTPD HTL PeLEDs and that detectable Au accumulation on the cathode is reduced by swapping the HTL for our mixed‐polymer. However, it is possible that this is due to reduced Au^+^ back‐transport through the mixed film, not that the anode corrosion is reduced. To directly confirm that our mixed‐polymer HTL is interfering with Au corrosion specifically, we developed a novel method to detect the presence of triiodide through a current‐based Au‐corrosion test. Based on Ag‐fuse tests introduced by Kerner et al. [14] and developed further by Hong et al. [24], these experiments use a metallic fuse exposed to a corrosive agent (here, iodine vapor) with a constant low current to monitor its conductivity. Iodine gas is used in lieu of perovskite so that we can use a diffusion gradient instead of an electrical bias to motivate transport through the HTL being tested. As polyTPD is known to be capable of reducing I_2_ to I^−^ and I_3_^−^, and I_2_ is an expected degradation product for MAPbI_3_, this assay simplifies the experiment while still mimicking the chemical environment the anode is exposed to as halides leak from the perovskite during voltage biasing. For example, this allows us to only supply and measure the test current without also supplying a voltage bias vertically through the sample. As iodine reacts with the metal to produce poorer‐conducting products at the reaction site, or if the metal is oxidized and liberated from the fuse, we measure a drop in conductivity. Figure 6a shows the general experimental setup for I_2_ atmosphere tests adapted for Au electrode testing. Two indium tin oxide (ITO) electrodes are bridged by a 35 nm Au fuse, which is bonded to the glass substrate by a 2 nm layer of Cr (details for improving the reliability of the fuse design are discussed in the Supporting Information, see Figures S5 and S6). Au is not oxidized by I_2_, so a decrease in conductivity across the two ITO electrodes can be assumed to be due to triiodide corrosion at the Au surface. The fuse is laminated with various HTL layers to discern how well they protect against Au corrosion in a saturated, gaseous I_2_ atmosphere. We improved the repeatability of our iodine corrosion tests by including 100 nm layers of evaporated bismuth that protect the areas where the Au fuse overlaps the 150 nm thick ITO electrodes. These overlap regions are less planar and can be preferentially corroded, reducing the repeatability of our test. By including Bi we prevent I_2_ interaction with these regions as well as standardize the exposure window of our Au fuse to a repeatable 3 mm × 17 mm window. Bismuth does not readily react with iodine, proving to be a chemically inert semimetal that has shown success protecting a perovskite solar cell from iodine corrosion [25]. The formation of BiI_3_, for example, from elemental Bi and I is endothermic and requires temperatures of 150°C [26], well beyond our room temperature experiment. To experimentally verify that Bi completely blocks I_2_ corrosion of sublayers, we created a 15 nm Au fuse sample that was completely covered by 50 nm of Bi and measured its conductivity in an I_2_ atmosphere for over 7 h, measuring no appreciable change in current (see Figure S7). Additionally, we tested the change in conductivity of the HTL films themselves when doped by I_2_ gas using the setup given in Figure 6b. Closely interdigitated Au electrodes on the HTL surface (150 µm separation with 0.5 cm electrode overlap) measure any increase in conductivity owing to the films’ uptake of iodine from a saturated atmosphere.

Under exposure to an I_2_ atmosphere at room temperature, a loss of conductivity of the Au fuse was detected over the course of 1 h for our pure polyTPD films and our mixed films with minimal inclusion of PVK (0.2 mg/mL). The other mixed films, pure PVK, and the no‐HTL control showed a roughly constant current level throughout the test (Figure 6c). Films that did not show obvious degradation even displayed a slight increase in current over the course of the experiment. This increase was due to a combination of the increase in conductivity of the HTL films as they absorb and are doped by I_2_, while iodine adsorbing on the fuse surface also increased overall sample conductivity. These results are consistent with an interference with triiodide transport and corrosion that we observe in stabilized top emitting PeLEDs.

Figure 6d shows the increased conductance of our HTL films in response to the presence of I_2_. A pure PVK film experiences no uptake of I_2_, leaving conductance at the noise floor of 10^−8^ S. All films with polyTPD experience a dramatic increase in conductance, plateauing in the first 100 s of exposure. The saturation conductance of the pure polyTPD film is an order of magnitude greater than the mixed films, indicating some level of I_2_ rejection occurring in the latter. We can be confident that the top surface of the mixed films are not pure PVK from our UPS DOS data and contact angle measurements (Figure 3a and Figure S4, respectively), and the rejection of iodine gas in our conductance test makes little sense if the PVK is spontaneously separating as a pure film on the buried interface of the deposited film. We therefore suspect the mixed‐polymer film structure involves some level of PVK dispersion throughout the polyTPD matrix.

## 3 Conclusion

From our results, we conclude that iodine transport and triiodide formation are reduced in our mixed‐polymer HTL due to the presence of relatively small amounts of the deeper‐HOMO level material PVK in the largely polyTPD layer. The HTL fabrication involves a simple single‐step spin‐coating and annealing process, which avoids the difficulties involved in forming bilayer solution‐processed HTLs. From UPS and contact angle measurements, we have demonstrated that the layer surface is dominated by polyTPD, maintaining high‐quality perovskite growth and hole injection. We detect Au accumulation at the cathode for our half‐PeLEDs, migration which requires a nonzero electrical bias, and show the rate of Au accumulation is reduced for our mixed layer compared to pure polyTPD, while the onset voltage of Au accumulation is increased for the mixed‐polymer. Our Au‐corrosion tests show that the presence of PVK mitigates the formation of triiodide in our polyTPD films and prolongs the life of the electrode layer over the course of an hour. Additionally, our conductivity tests show that the presence of PVK reduces the film's susceptibility to iodine‐induced doping in a saturated gaseous atmosphere. The improved stability demonstrated in MAPbI_3_ LEDs (Figure 2) can thus be reasonably linked to reduced halide corrosion of the Au anode as the HTL interferes similarly with solid‐state iodine transport from the emission layer. Additionally, we present the first experimental test to quantify the corrosion of an anode by triiodide, which is generalizable to future tests for further HTL refining. Future efforts can use Au‐corrosion tests to identify longer‐term iodine rejecting HTL films and better establish how the choice of the HOMO level and quantity of the added polymer can affect the lifetime of metal anodes in PeLEDs. As perovskite optoelectronic devices continue to improve in efficiency the simultaneous need for improved stability will require deeper analysis of the charge transport layers and their role in maintaining high‐quality perovskite crystal growth, carrier injection, and reduce deleterious reactions that source from the perovskite layer itself.

## 4 Methods

### 4.1 Perovskite LEDs

Perovskite precursor solutions consisted of lead iodide (PbI_2_, TCI, 99.99% purity), methylammonium iodide (MAI, Greatcell Solar Materials, > 99.99% purity), and benzylmethylammonium iodide (BzAI, Greatcell Solar Materials, > 99% purity) dissolved in N,N‐dimethylformamide (DMF, Sigma–Aldrich, anhydrous 99.8%). All precursors were dissolved in DMF at 0.8 m, and PbI_2_ required heating and stirring overnight to fully dissolve. Precursors were combined immediately preceding fabrication in a ratio of 5:5:1 PbI_2_:MAI:PMAI, with DMF added to reduce the concentration to 0.2 m MAPbI_3_. HTL precursors were prepared from poly[N,N’‐bis(4‐butylphenyl)‐N,N’‐bis(phenyl)‐benzidine] (polyTPD, American Dye Source, Inc., Hole Transport Polymer ADS254BE) and poly(9‐vinylcarbazole) (PVK, Sigma–Aldrich) dissolved in chlorobenzene (CB, Sigma–Aldrich, anhydrous 99.8%). Stock solutions were filtered using a PTFE filter from VWR, 0.45 µm. Filtered solutions were combined according to reported ratios. Due to the complexity in producing precise concentrations from two different stock solutions, actual concentration ratios differ slightly from the target concentrations. By starting with a 6 mg/mL polyTPD solution and adding small amounts of 6 mg/mL PVK solution, we achieved approximations to the target concentration ratios. Table 1 accounts for the increase in volume from the added PVK solutions and translates the exact concentrations used for the target ratios referenced in the text. Additionally, Table 1 shows layer thicknesses of standalone HTL films grown on Si/SiO_2_ substrates using the same solution concentrations and spin‐coating conditions as described. HTL film thicknesses were measured using a Woollam M‐2000 spectroscopic ellipsometer. “0.8 PVK” mixture is included for completeness as this mixture was studied using UV–Vis and AFM (see Figure 3b and Figure S4), but was not used in PeLED devices and did not have its standalone thickness measured.

For top emitting PeLEDs, 3 cm × 3 cm soda‐lime glass substrates were sequentially cleaned with detergent, deionized water, acetone, and isopropyl alcohol for 15 min each in a sonication bath, followed by a 15 min O_2_ plasma treatment. Onto these Cr (2 nm bonding layer), Au (100 nm), and molybdenum oxide (MoO_3_, Alfa Aesar Puratronic, 99.9995% purity, 10 nm) were evaporated in an Angstrom Engineering EvoVac system at 1 µTorr. For bottom emitting PeLEDs, 3 cm × 3 cm soda‐lime glass substrates (0.7 mm thick) with commercially patterned ITO (150 nm, < 15 Ω/sq.) were purchased from Colorado Concept Coatings LLC and subject to the same cleaning procedure detailed above. Spin‐coating the HTL solution at 1000 rpm for 45 s formed a roughly 20 nm thick layer, followed by thermal annealing for 20 min at 150°C. A 15 s O_2_ plasma treatment improved wettability for the following perovskite layer (6000 rpm for 35 s; solvent quench after 5 s with toluene (Sigma–Aldrich, anhydrous 99.8%), 30 nm final layer thickness). Samples were then annealed at 70°C for 5 min. In vacuum were subsequently deposited 40 nm of 2,2',2''‐(1,3,5‐benzinetriyl)‐tris(1‐phenyl‐1‐H‐benzimidazole) (TPBi), a bilayer of LiF (1.2 nm) and Al (2 nm), and, for top emitting PeLEDs, a top‐electrode of Ag:Al alloy (15:1 volumetric ratio) with ∼9 nm thickness (substrate cooling to 0°C and rapid evaporation rates were used to improve thin‐film growth of the alloy). For bottom emitting PeLEDs, the Al portion of the LiF/Al bilayer was increased to a total of 100 nm. Electrode overlap defined PeLED active areas of 10 mm^2^ (4 × 2.5 mm^2^) for bottom emitting devices and 2.5 mm^2^ (1 × 2.5 mm^2^) for top emitting devices. *J–V* curves were measured using a Keithley 2400 SourceMeter. LED output was measured using a calibrated Si photodiode (FDS‐100‐CAL, Thorlabs), measured by a picoammeter (Agilent 4140B) to quantify LED emission intensity. Angular emission profiles were collected with the photodiode on a homemade goniometer setup to calculate external quantum efficiencies. LED EL spectra were measured using a spectrophotometer (UVN‐SR, StellarNet Inc.).

### 4.2 Au‐Migration XPS, SEM, and EDX

The ITO/MAPbI_3_/HTL/Au samples for XPS, SEM, and EDX measurements for the study of voltage‐induced Au migration across different HTLs were fabricated on patterned ITO substrates (same as used for bottom emitting PeLEDs above) that were sequentially cleaned with diluted detergent, deionized water, acetone, and isopropanol for 10 min each under sonication, followed by a 10 min O_2_‐plasma treatment prior to perovskite deposition. Perovskite precursor solution (0.8 m PbI_2_ + 0.8 m MAI in DMF) was spin‐coated on the cleaned ITO substrates in a N_2_‐filled glovebox via a two‐step program: 1000 rpm for 10 s followed by 6000 rpm for 30 s. 100 µL of CB was dropped onto the substrate as an antisolvent 25 s before the end of the spin‐coating program. The samples were immediately annealed on a 100°C hotplate for 30 min. HTL precursor solutions (polyTPD, PVK, or “0.4 PVK”) were then spin‐coated on the perovskite layer under a spin rate of 1000 rpm for 45 s. The devices were completed by thermal evaporation of 100 nm of Au through a shadow mask defining 12 pixels per substrate, each with an active area of ∼0.1 cm^2^ (4 × 2.5 mm^2^).

The buried interface of the electrode was exposed by physically delaminating the Au electrode using a conductive carbon tape. The HTL and perovskite layers were washed off with CB and DMF sequentially to expose the ITO interface.

ITO/MAPbI_3_/HTL/Au devices were voltage‐biased in a N_2_‐filled environment in the dark at room temperature using a Keithley 2602B SourceMeter controlled by SCPI Matlab scripts. Scanning electron microscopy (SEM) images and energy‐dispersive x‐ray spectroscopy (EDX) were measured by an FEI Verios 460 XHR SEM. Samples were coated with 3 nm of iridium before SEM measurements. X‐ray spectroscopy (XPS) measurements were performed by a Thermo‐Scientific Kα x‐ray Photoemission Spectrometer operating at a base pressure of < 1 × 10^−7^ mbar and using an Al anode at a power of 72 W.

### 4.3 Au‐Fuse Samples

Au‐fuse samples were created from commercially available 3 cm × 3 cm soda‐lime glass substrates with indium tin oxide electrodes pre‐deposited (150 nm). Cr (2 nm) and Au (35 nm) were thermally evaporated in a 17 mm × 17 mm square centered on the substrate using a shadowmask in an Angstrom Engineering EvoVac system at 1 µTorr. Bi (100 nm) is subsequently thermally evaporated on top of the Au fuse using a shadowmask that carefully leaves a 3 mm × 17 mm window centered between the Bi layers for I_2_ exposure.

After Au‐fuse samples are evaporated, they can be subject to solution‐processed deposition of various HTL materials or left exposed bare for a control sample. HTL materials can alternatively be evaporated onto the Au‐fuse surface.

### 4.4 Conductance Measurement Samples

Samples for the conductance measurement involve depositing the HTL on a 3 cm × 3 cm soda‐lime glass substrate using spin‐coating and annealing. Interdigitated electrodes are evaporated using Au (100 nm) in an Angstrom Engineering EvoVac system at 1 µTorr onto the surface of annealed HTL films. The electrodes are separated by 150 µm spacing over 0.5 cm of interdigitation overlap.

### 4.5 I_2_ Exposure Tests

A wide‐necked flask containing pure I_2_ crystals is sealed with parafilm and left for at least 15 min at room temperature to allow saturation of I_2_ gas inside the beaker, confirmed by the presence of uniform purple gas. Au‐fuse samples and conductance measurement samples are connected to a Keithley 2400 SourceMeter using alligator clips. The beaker is sealed using parafilm with the samples inside. The beakers are kept in a dry box constantly purged with dry N_2_. Samples are tested with 0.1 V, with current recorded for variable testing periods using a MATLAB script.

### 4.6 Film Characterization

Film absorbance and transmission were measured using an Agilent Cary 5000 UV–Vis–NIR spectrometer. Baseline transmission/absorbance was established using a cleaned and bare soda‐lime glass substrate. Film morphology was measured using a Bruker Dimension ICON3 Atomic Force Microscope. Contact angle measurements were performed using a ramÉ‐hart model Goniometer using a drop of deionized water in ambient. DROPimage Pro was used to calculate the contact angle from collected images. 3 separate water drops on different areas of each sample were analyzed for each HTL layer for statistics (3 cm × 3 cm glass substrate underlayer). Scanning electron microscopy (SEM) images of Au‐fuse morphology were characterized by an FEI Verios 460 XHR SEM. Samples were coated with 3 nm of iridium before SEM measurements. UPS measurements were conducted using He I (He II) radiation with photon energies of 21.22 eV (40.81 eV) on a Thermo Fisher Scientific Nexsa G2 system, with pass energies of 2 eV (10 eV) and a step size of 0.02 eV. All measurements were calibrated against gold and corrected for satellite emissions.

## Conflicts of Interest

The authors declare no conflicts of interest.

## Supporting information




**Supporting File**: advs76051‐sup‐0001‐SuppMat.pdf.

## Data Availability

The data that support the findings of this study are available from the corresponding author upon reasonable request.
