# Supplementary material for: Mixed‐Polymer Hole Transport Layers Reinforce Au‐Anode Top Emitting Perovskite Light Emitting Diodes
Source: Adv Sci (Weinh). 2026 Jun 11:e76051. Online ahead of print. doi: 10.1002/advs.76051 (PMC13336438; doi:10.1002/advs.76051)
Supplement: Supplementary file 1 — Supporting File: advs76051‐sup‐0001‐SuppMat.pdf. [file ADVS-9999-e76051-s001.pdf]

Supplementary Information for Mixed-Polymer Hole Transport Layers Reinforce Au-Anode Top Emitting Perovskite Light Emitting Diodes

James C. Loy[1], Jisu Hong[2], Tuo Hu[2], Xu He[2], Antoine Kahn[2], Barry P. Rand[2,3]

[1]Department of Physics, Princeton University, Princeton, NJ, 08544 USA

[2]Department of Electrical and Computer Engineering, Princeton University, Princeton, NJ, 08544 USA

[3]Andlinger Center for Energy and the Environment, Princeton University, Princeton, NJ, 08544 USA

Supplementary Information

Seven figures, one table, and a discussion on improving repeatability of Au-fuse samples used in I_2 vapor experiments.

Bottom Emitting Perovskite Light-Emitting Diode (PeLED) on ITO

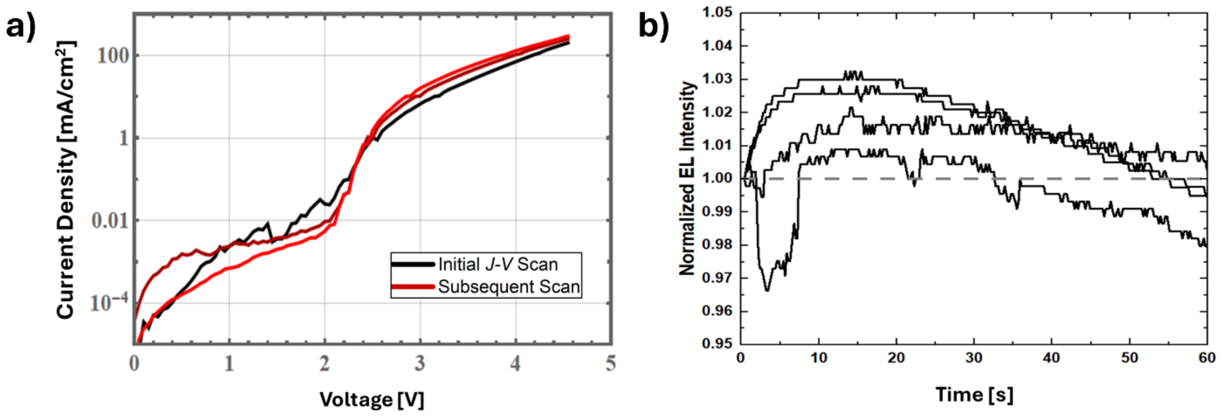

Figure S1: (a) Bottom emitting perovskite LED *J*-*V* characteristics (pure polyTPD HTL) under repeated voltage sweep, showing a much more repeatable *J*-*V* curve for a single pixel than the top emitting PeLED analogue, shown in Figure 1 of the main text. Consecutive LED operation shown by increasingly lighter red scans. (b) Under constant operation at a fixed current density of 5 mA/cm^2, emission intensity is essentially unchanged over the course of 60 s. Each trend represents a distinct but nominally identical pixel. Both degradation signatures seen in the top emitting device on Au-anode are absent from the corresponding bottom emitting device on an ITO-anode.

Top Emitting PeLED Characterization

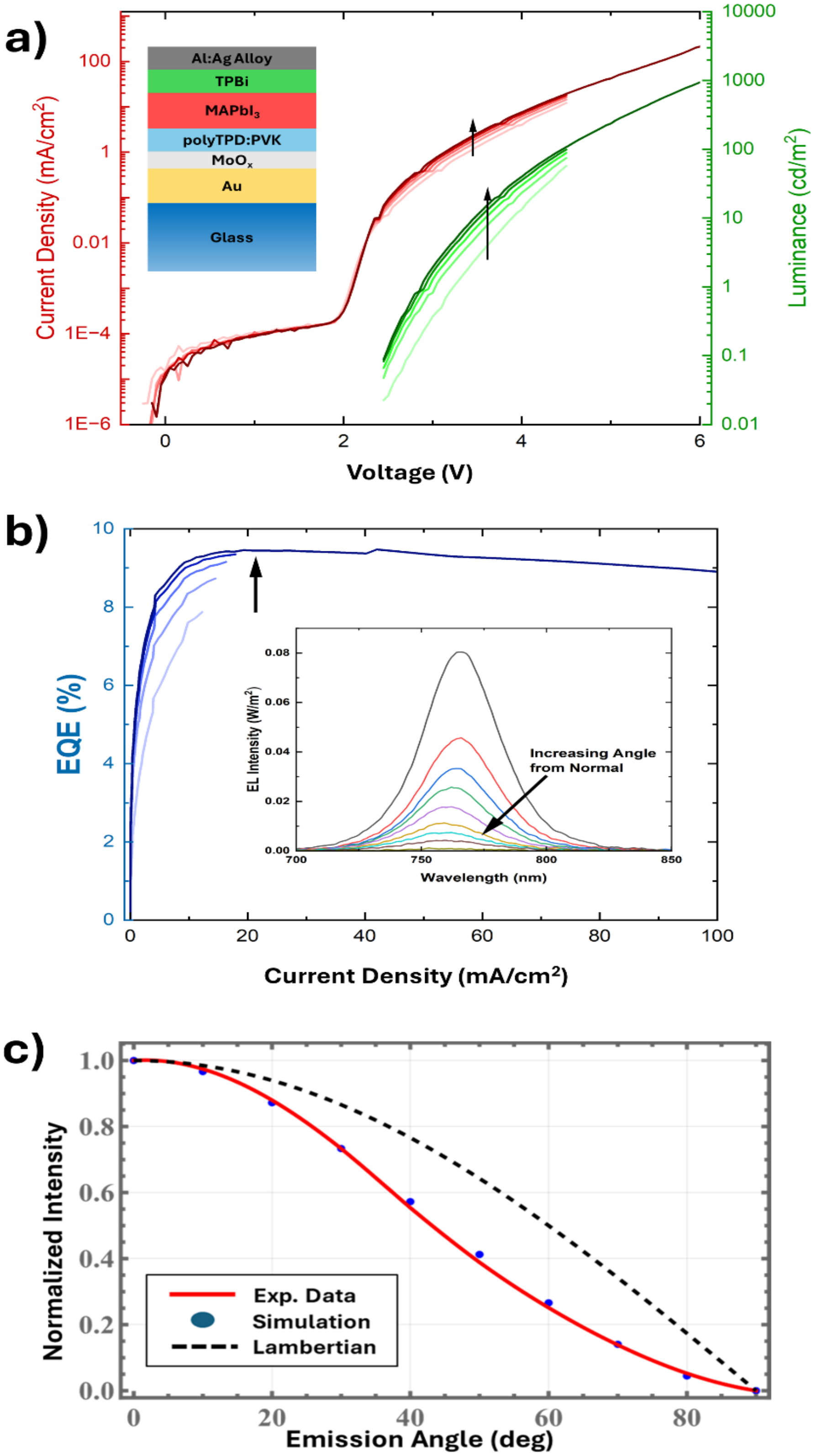

Figure S2: Top emitting $MAPbI_3$ perovskite LED typical performance characteristics (when stabilized with a mixed-polymer hole transport layer). (a) Current density-voltage-luminance (*J-V-L*) data for a representative pixel under repeated voltage scans shows a typical turn on voltage near 2.3 V, a maximum current density above 100 mA/cm^2, and a maximum luminance of 1000 cd/m^2 at 6 V during the final voltage scan. Black arrows show steady improvement in performance with repeated scans. *inset:* PeLED stack structure. (b) External quantum efficiency (EQE) is calculated for each voltage scan and shows the same improvement trend seen in the *J-V-L* plot. The final scan shows low roll-off of the maximum efficiency up to 100 mA/cm^2. *inset:* The LED electroluminescence (EL) spectra have a full width at half maximum (FWHM) of 37 nm and centers on 766 nm emission peak. The peak blue-shifts slightly with increasing emission angle due to the planar microcavity formed by the reflective and opaque Au anode and the semi-transparent Ag/Al alloy cathode, shifting to 759 nm for 60° viewing angle. Spectra were collected in a black box at 10° intervals from 0° to 80° with constant current density of 40 mA/cm^2. (c) The pixel's angular profile shows a strong forward output consistent with the planar microcavity (recorded under constant current density of 5 mA/cm^2). Simulation values were calculated using a transfer-matrix method based on the refractive index and layer thicknesses of the LED. See Methods in the main text for data-collection setup details.

Surface Morphology Analyses of HTLs

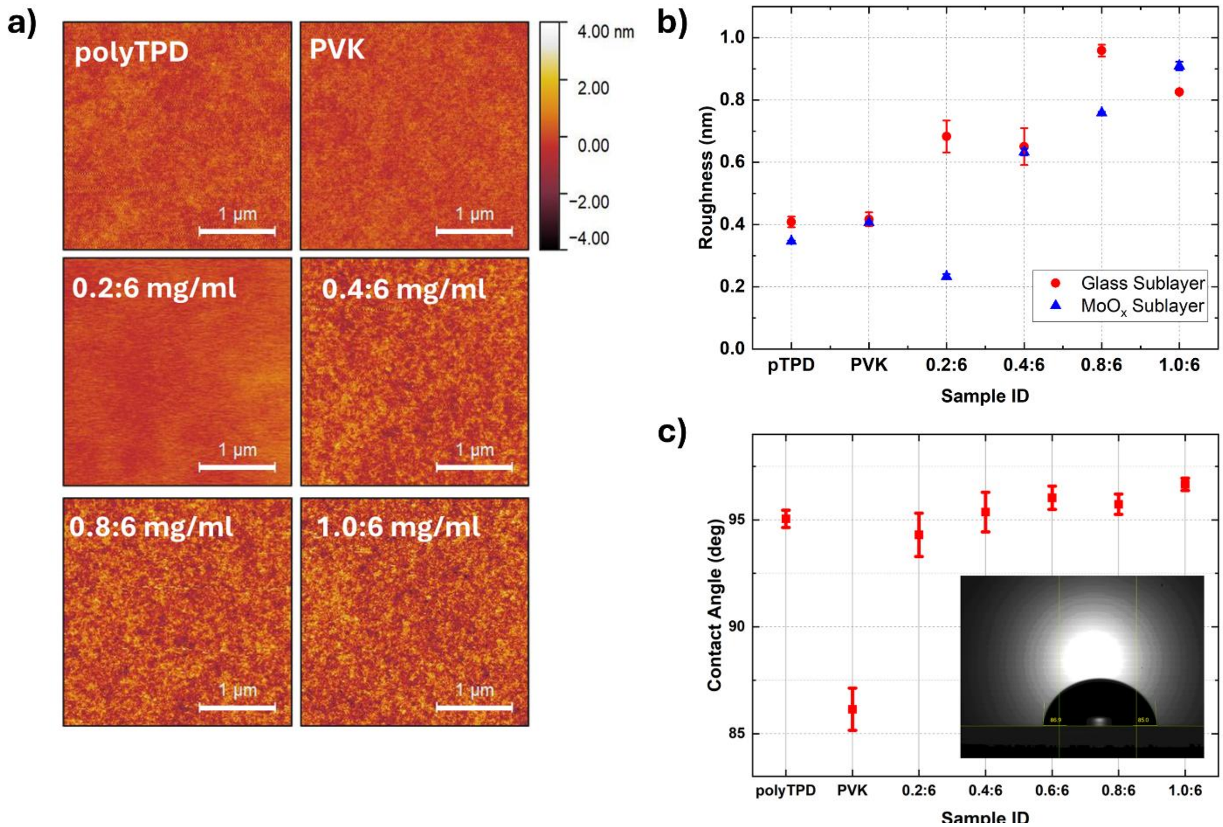

Figure S3: HTL surface analyses. (a) AFM images of the surface of HTL films with (b) corresponding roughness measurements for various mixed HTL films (with and without a MoO_x sublayer on glass). Roughness increases slightly for mixed films, but generally all films are smooth and pinhole free. Ratios given are mg of PVK and polyTPD respectively per ml of chlorobenzene solvent in all subplots. (c) Contact angle measurements for various HTL films using deionized water in air. PVK and polyTPD are distinguishable (over 7° difference) in this experiment. All mixed films correspond strongly with polyTPD, indicating it dominates the top surface (contact angle is determined by short-range van der Waals forces). Error bars are standard deviation of measurements for 3 separate drops on different areas of the sample surface. *inset*: water droplet on a pure PVK film.

HTL UPS Spectra

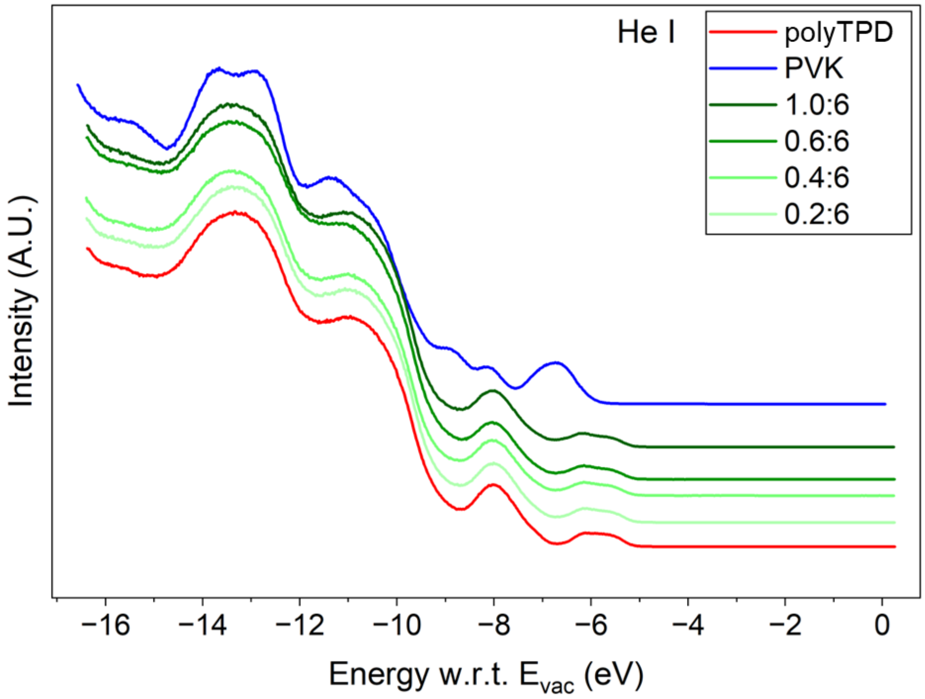

Figure S4: He I ultraviolet photoelectron spectra (UPS) of neat PVK, polyTPD, and mixed-polymer films. Mixed film spectra are overwhelmingly similar to pure polyTPD in spectra shape, even for the highest PVK loading (1.0:6).

Table S1: Extracted HOMO level, onset, work function (WF), and ionization energy (IE) from He I UPS measurement (see Figure S4). PVK and polyTPD are distinct in each measurement, while the mixed films strongly favor polyTPD in every instance. Raw data reported to 0.01 eV precision to emphasize this similarity, but resolution is ±0.1 eV for each measurement.

polyTPD:PVK	HOMO	onset	WF	IE
**PVK**	1.36	16.66	4.56	5.92
**polyTPD**	0.76	16.86	4.36	5.12
**1.0:6**	0.73	16.84	4.38	5.11
**0.6:6**	0.73	16.85	4.37	5.10
**0.4:6**	0.73	16.84	4.38	5.11
**0.2:6**	0.74	16.85	4.37	5.11

Improving Au-Fuse Repeatability

To maximize the current-based signal in our Au-fuse tests, the corroded portion of the Au-fuse must be significant relative to the cross-sectional area of the fuse, requiring the Au to be as thin as possible. For thermal evaporation, we found a complete (i.e., percolated) film requires a minimum 10 nm thickness, with sample yield improving significantly at 15 nm Au. However, when testing samples with this layer thickness in I_2, we observed spontaneous current loss at random times with an instant drop to zero current, inconsistent with our predicted steady, gradual corrosion process. We identified the Cr bonding layer between our Au-film and glass substrates as the issue. CrI_3 is readily formed when I_2 reacts with metallic Cr, leading to selective corrosion of the bonding layer through the incomplete Au layer. This process disrupts the Au film and leads to an open-circuit condition, with the lifetime of the fuse depending on how easily I_2 can penetrate the Au film rather than how quickly the lixiviant reaction occurs.

Removing Cr entirely from the samples was impossible due to spontaneous delamination that Au suffers from when bonded directly to glass (Figure S5), and no suitable I_2 resistant bonding material could be identified. Using scanning electron microscopy (SEM), we found that our Au deposition demonstrated island-growth dominated films for thicknesses below 35 nm. Complete films were observed at thicknesses around 35 nm and above (see Figure S6). Using 35 nm thick Au-fuses we were then able to discern gradual conductivity changes without spontaneous open-circuit formation, indicating the gradual Au-corrosion process expected for Au films exposed to I^- and I_3^-. This signal verified that the uniform Au film was successfully protecting the Cr layer beneath from corrosion over the course of our experiment.

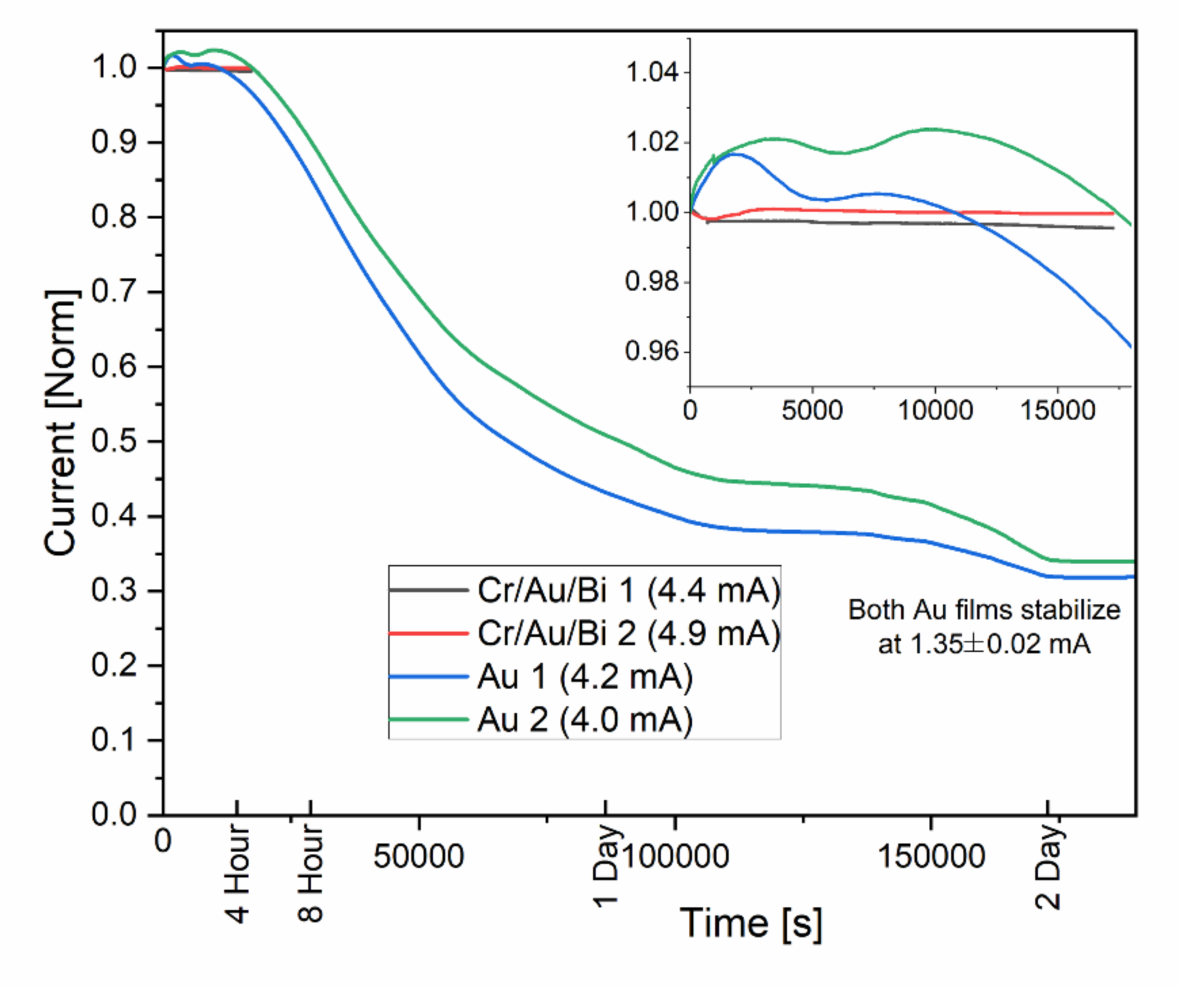

Figure S5: Spontaneous delamination of Au fuse in ambient (no I_2 exposure). Two samples of 15 nm of Au on glass with no bonding layer were compared to two samples with a 2 nm Cr bonding layer between the Au and glass. Over the course of 2 days, significant current drift indicates some thin-film evolution during that period in the non-bonded layer. With a Cr bonding layer added, samples show a stable current over 4 hours of testing. Inset: while the Cr bonding layer samples were not left for 2 days, it is clear when comparing the initial 4 hours that the bonding layer samples are unmatched for constant conductivity, indicating that spontaneous delamination is not only occurring during this period in the nonbonded samples, but is a dominant mechanism for changes in conductivity observed in films with no bonding layer.

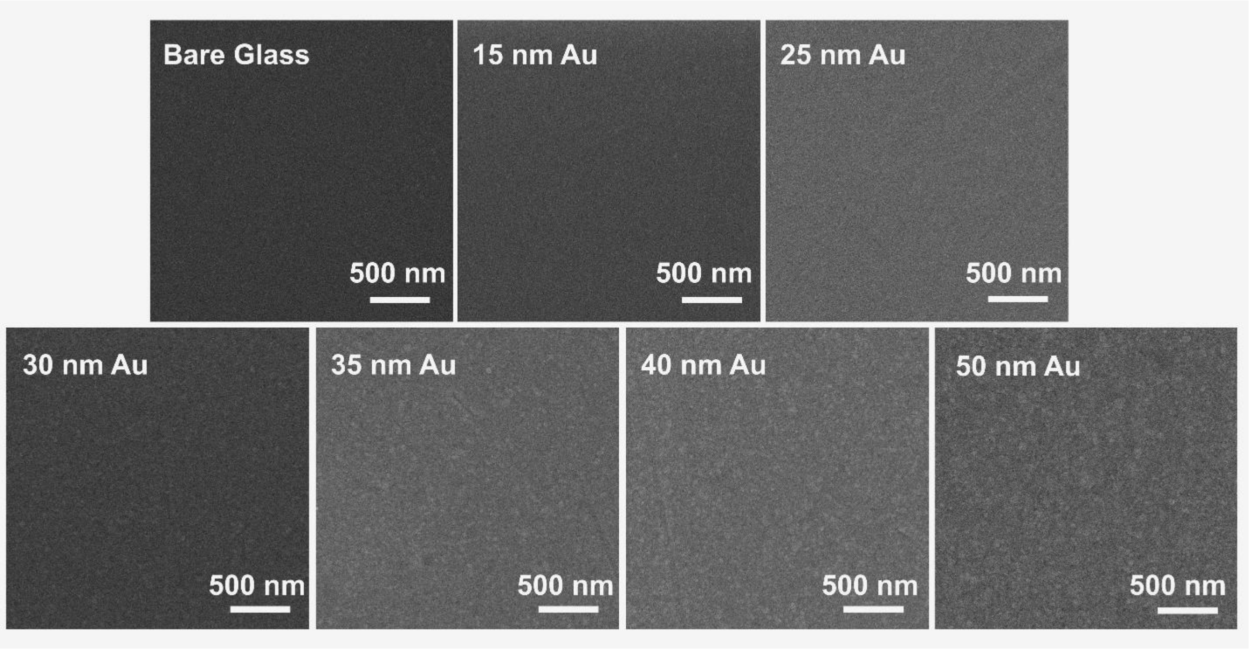

Figure S6: SEM images of Au films on Cr bonding layers for various Au thicknesses. The Au does not appear to form a complete film below 35 nm thickness. Image contrast has been adjusted for clarity.

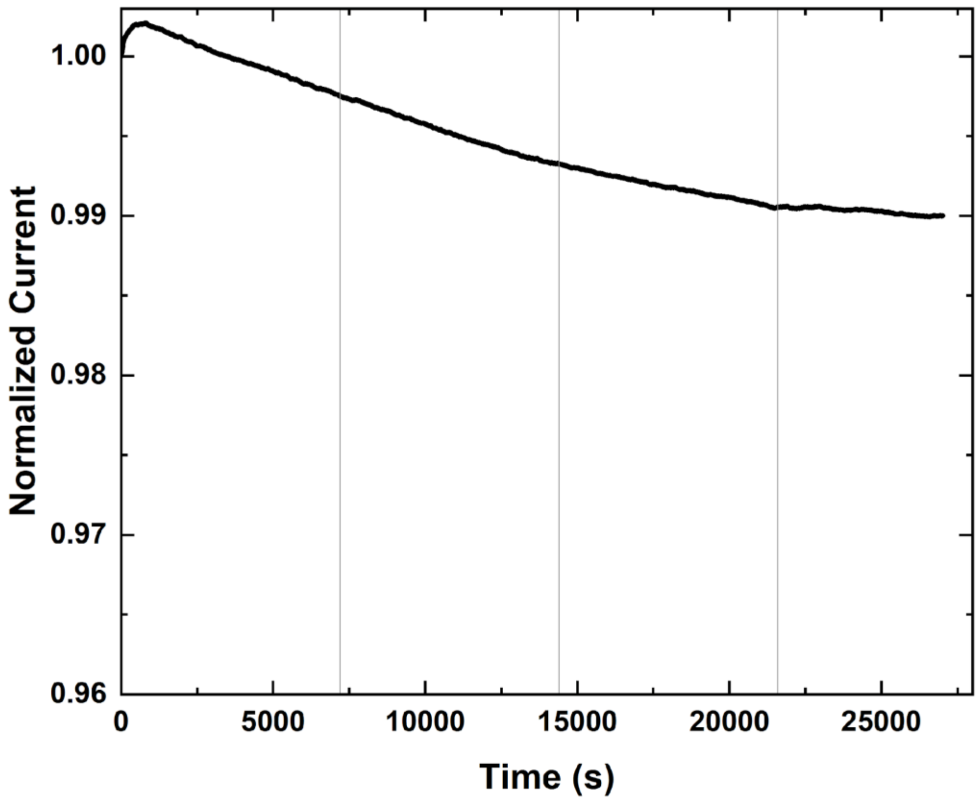

Figure S7: 15 nm Au-fuse sample (with 2 nm Cr bonding layer) in I_2 atmosphere with a 50 nm uniform, evaporated bismuth layer covering the entirety of the Au fuse. 1% of initial current is lost over the course of almost 8 hours. Vertical reference lines are spaced 2 hours apart.
